# Health Outcomes of Caregiver-Friendly Workplace Policy Interventions: A Systematic Review

**DOI:** 10.1093/geroni/igaf122.2157

**Published:** 2025-12-31

**Authors:** Maryssa Pallis

**Affiliations:** University of Massachusetts Boston, Boston, Massachusetts, United States

## Abstract

Support for caregiver-friendly workplace policies (CFWPs) is on the rise. Recent attention has grown around workplace interventions of CFWPs and related health outcomes. However, little research has been done synthesizing findings across varying intervention types. This systematic review aimed to identify CFWP intervention studies, comparing the methods, types of interventions, and outcomes related to caregiver health. The search, conducted in December 2024, originally yielded 25 articles overviewing a CFWP intervention. Following eligibility screening, 10 articles were included in the final review. In terms of methods, six were randomized control trials (some cluster-randomized control trials and some random assignments to a waitlist control condition), three were pre-and post-tests, and one was a pilot study. From low to high-quality intervention studies, results showed improvements in physical health (cardiometabolic risk factors, self-rated health, and sleep) and mental health (depression, anxiety, and self-efficacy) post-implementation of CFWPs. Across intervention types and study design variations, CFWP interventions were associated with improvements in both physical and mental health. Evidence points to CFWPs being health-promoting, with worksites offering new capabilities for advancing the health of caregivers managing dual roles of work and informal caregiving responsibilities. Findings also point to the need for additional intervention studies examining CFWPs and increased coordination around methods and intervention types.

